# Poly ADP Ribose Polymerase Inhibitor Olaparib Targeting Microhomology End Joining in Retinoblastoma Protein Defective Cancer: Analysis of the Retinoblastoma Cell-Killing Effects by Olaparib after Inducing Double-Strand Breaks

**DOI:** 10.3390/ijms221910687

**Published:** 2021-10-01

**Authors:** Yuning Jiang, Jason C. Yam, Wai Kit Chu

**Affiliations:** 1 Department of Ophthalmology & Visual Sciences, The Chinese University of Hong Kong, Hong Kong 999077, China; xdk4xz@virginia.edu (Y.J.); yamcheuksing@cuhk.edu.hk (J.C.Y.); 2 Department of Radiation Oncology, University of Virginia, Charlottesville, VA 22908, USA; 3 Hong Kong Hub of Paediatric Excellence, The Chinese University of Hong Kong, Hong Kong 999077, China

**Keywords:** PARP inhibitor, olaparib, etoposide, DNA double-strand break repair, retinoblastoma

## Abstract

Retinoblastoma is the most common intraocular cancer in childhood. Loss of function in both copies of the *RB1* gene is the causal mutation of retinoblastoma. Current treatment for retinoblastoma includes the use of chemotherapeutic agents, such as the DNA damaging agent etoposide, which is a topoisomerase II poison that mainly generates DNA double-strand breaks (DSBs) and genome instability. Unfaithful repairing of DSBs could lead to secondary cancers and serious side effects. Previously, we found that RB knocked-down mammalian cells depend on a highly mutagenic pathway, the micro-homology mediated end joining (MMEJ) pathway, to repair DSBs. Poly ADP ribose polymerase 1 (PARP1) is a major protein in promoting the MMEJ pathway. In this study, we explored the effects of olaparib, a PARP inhibitor, in killing retinoblastoma cells. Retinoblastoma cell line Y79 and primary retinoblastoma cells expressed the cone-rod homeobox protein (CRX), a photoreceptor-specific marker. No detectable RB expression was found in these cells. The co-treatment of olaparib and etoposide led to enhanced cell death in both the Y79 cells and the primary retinoblastoma cells. Our results demonstrated the killing effects in retinoblastoma cells by PARP inhibitor olaparib after inducing DNA double-strand breaks. The use of olaparib in combination with etoposide could improve the cell-killing effects. Thus, lower dosages of etoposide can be used to treat retinoblastoma, which would potentially lead to a lower level of DSBs and a relatively more stable genome.

## 1. Introduction

Retinoblastoma is the most common intraocular cancer in childhood. It accounts for approximately 3% of all childhood cancer [1]. The prevalence of retinoblastoma is approximately 1 in 15,000 live births. Eight thousand children are diagnosed with retinoblastoma each year worldwide [2]. Retinoblastoma patients usually show signs of leukocoria or strabismus. The median age of presentation is around 18 months. Retinoblastoma is a tumor occurring in the retina. It has been reported that retinoblastoma originates in the cone photoreceptor precursor cells [3]. Loss of both copies of the retinoblastoma (*RB1)* gene on chromosome 13q 14 is thought to be the most common cause of retinoblastoma [4]. In 1971, the “two-hit” mutation hypothesis was proposed to explain the cause of retinoblastoma [5], and the *RB1* gene was identified 16 years later [6]. *RB1* is the first tumor suppressor gene identified whose mutational inactivation would lead to the formation of a tumor [7]. Functional disruption of *RB1* can be generated by the somatic inactivation of both *RB1* alleles, or with a germline *RB1* mutation in one allele and a somatic inactivation of the second *RB1* allele. This is described by the famous “two-hit hypothesis” to suggest the genetic mutation of both alleles of a single gene would lead to tumorigenesis [8]. In addition, inactivation of the *RB1* gene could also happen in DNA sequence alteration, promoter methylation, and loss of heterozygosity [9]. Importantly, the risk of a second malignancy is very high in retinoblastoma patients. It was reported that during a 50 year period, around 46% retinoblastoma survivors developed a secondary cancer [10]. Current treatment for retinoblastoma aims to save the lives of patients, prevent the loss of eyes, and preserve useful vision. Depending on different clinical stages of retinoblastoma, the tumor may be confined to the retina (stage A–B), vitreous seeding may occur (stage C–D), or the condition may be unsalvageable (stage E) [11]. For intraocular retinoblastoma, several local treatments, such as laser, cryotherapy, radiation, and chemotherapy, are commonly used. At very late stages, enucleation to remove the eye is needed in order to save the life of the patient. For extraocular retinoblastoma, intensive chemotherapy, such as etoposide, is required [2]. Etoposide is a type II topoisomerase poison. Etoposide could generate DNA double-strand breaks (DSBs), which is considered the most toxic DNA damage due to the complete discontinuation of genetic materials. The severe side effects of chemotherapy, such as elevated risk of secondary cancer, including osteosarcoma and leukemia, is commonly observed in retinoblastoma patients [12]. In addition, diminished orbital growth, hearing loss, and enucleation would lead to a very low quality of life in patients.

The major cause of retinoblastoma was originally thought to be a consequence of mutations on both *RB1* alleles [5]. Recent work has reported that the retention of a single functional *RB1* allele is insufficient to maintain genome stability. Loss of one functional *RB1* allele results in DNA replication stress, accumulation of DSBs, mitotic errors, and dysregulation of p53 functions. The term “haploinsufficiency” is used to described the dosage effect in which the wild-type RB protein, generated from the single wild-type allele, is not sufficient to fulfil its functional requirements [13].

Recently, we reported that RB knocked-down U2OS cells could repair DSBs by employing a highly mutagenic micro-homology mediated end joining (MMEJ) pathway [14]. MMEJ is an error-prone DNA repair pathway. Several proteins, such as poly ADP ribose polymerase 1 (PARP1), polymerase theta (POLQ), and DNA ligase 3, are important regulators in MMEJ. In addition, our findings are consistent with previous studies of RB functions in promoting other DSB repair pathways, homologous recombination (HR), and canonical non-homologous end joining (C-NHEJ) [15,16]. Interestingly, previous research has sequenced flanking deletion breakpoints in the *RB1* gene in retinoblastoma tumors and found 4 to 7 bp direct repeat deletions. This suggests that the MMEJ pathway may be employed to repair DSBs in retinoblastoma cells [17]. Similar to our findings, some other cancers, such as breast cancer, are also defective in HR and could be treated with a PARP inhibitor [18]. Based on these observations, we evaluated the killing effects of the PARP inhibitor olaparib in retinoblastoma cells after inducing DSBs. 

## 2. Results

### 2.1. RB Deficient Cells Depend on MMEJ to Repair DSBs and Are Hypersensitive to Etoposide Treatment

**Figure 1:**
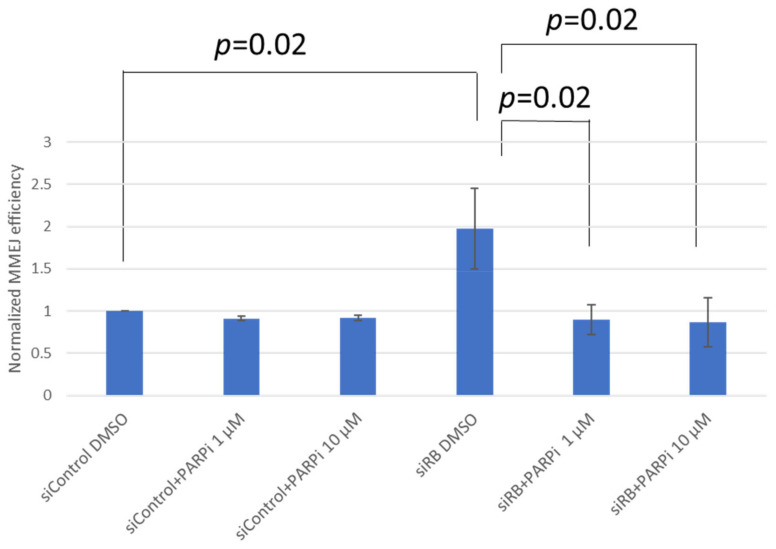
MMEJ efficiency was measured in non-targeting control siRNA (siControl) and RB siRNA (siRB)-transfected U2OS cells. Cells were also treated with DMSO, 1 μM or 10 μM olaparib (PARPi) for 1 h. Experiments were repeated three times. Data are shown as mean ± standard deviation and analyzed using two-tailed unpaired *t*-test.

**Figure 2:**
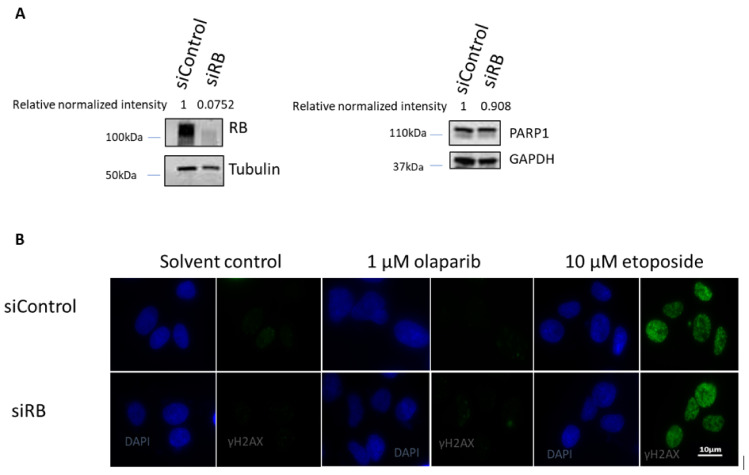
(**A**) RB was knocked-down efficiently three days after RB siRNA treatment, while PARP1 expression remained unchanged. GAPDH was used as the loading control. Western blot was repeated two times independently. Relative band intensities of RB and PARP1 are shown by normalizing with tubulin and GAPDH, respectively. (**B**) Immunofluorescence staining of γH2AX foci (green) in RB knocked-down cells (siRB) and control siRNA-treated cells (siControl) after treating with solvent control, 1 μM olaparib or 10 μM etoposide for one hour. Nuclei were stained with DAPI (blue). Immunofluorescence staining was repeated two times independently. Quantification of cells showing more than five γH2AX per cell is shown in Supplementary Figure S1.

In our previous study, we observed that MMEJ efficiency was elevated in RB knocked-down cells. Therefore, we hypothesize that the PARP inhibitor olaparib could impede MMEJ in RB knocked-down cells. Our results demonstrated that RB knocked-down cells could significantly enhance MMEJ, while a one-hour treatment of the PARP inhibitor olaparib had no effect on MMEJ. Interestingly, olaparib treatment was able to suppress MMEJ significantly in RB knocked-down cells, suggesting that PARP is important for the elevated MMEJ in RB knocked-down cells (Figure 1). Next, we studied the DSB levels in U2OS cells treated with etoposide or olaparib, after knocking-down RB. Obvious RB loss could be observed in *RB1* siRNA-treated U2OS cells. Equal expression levels of PARP1 were observed in RB knocked-down cells and control siRNA-treated cells, implying that RB loss would not affect the expression of PARP1 (Figure 2A). In both control siRNA-treated cells and *RB1* siRNA-treated cells, significant elevation in the number of γH2AX foci, an indicator of DSB levels, could be observed after etoposide treatment, while the PARP1 inhibitor did not induce additional DSBs (Figure 2B).

**Figure 3:**
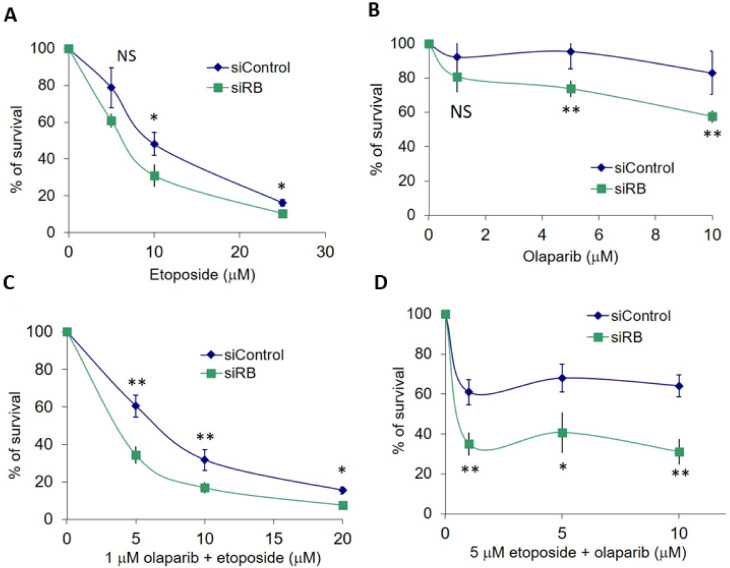
(**A**) MTT assay in RB knocked-down (siRB) and control siRNA (siControl)-treated U2OS cells showed cell survival after treating various dosages of etoposide for three days. (**B**) MTT assay in RB knocked-down and control siRNA-treated U2OS cells showed cell survival after treatment with various dosages of olaparib for three days. (**C**) MTT assay in RB knocked-down and control siRNA-treated U2OS cells showed cell survival after co-treatment with 1 μM olaparib with various dosages of etoposide for three days. (**D**) MTT assay in RB knocked-down and control siRNA-treated U2OS cells showed cell survival after co-treatment with 5 μM etoposide with various dosages of olaparib for three days. Experiments were repeated three times. Data are shown as mean ± standard deviation and analyzed using two-tailed unpaired *t*-test: * represents *p* < 0.05, ** represents *p* < 0.01, NS represents no significance.

Unrepaired DSBs could lead to cell death. Cell survival was quantified by the MTT assay. RB knocked-down U2OS cells were hypersensitive to etoposide treatment. Significantly less cell survival was observed in RB knocked-down cells at 10 µM and 25 µM etoposide (Figure 3A). Significantly enhanced killing effects of RB knocked-down cells were also observed after treatment with 5 µM and 10 µM olaparib (Figure 3B). Based on our results, we noticed that no significantly enhanced killing could be observed in RB knocked-down cells treated with 5 µM etoposide or 1 µM olaparib (Figure 3A,B). Therefore, we explored if the co-treatment of etoposide and olaparib could improve the insignificant killing effects at 5 µM etoposide and 1 µM olaparib. In our first experiment, we co-treated various concentrations of etoposide with 1 µM olaparib and observed significantly less cell survival in RB knocked-down cells (Figure 3C). Then, we co-treated various concentrations of olaparib with 5 µM etoposide. Significantly less cell survival could also be detected under these conditions in RB knocked-down cells (Figure 3D). Our results demonstrated that the co-treatment of etoposide and olaparib could improve the cell killing effects of single-drug treatments at dosages that had no significant effects on cell survival.

### 2.2. RB Expression in Primary Retinoblastoma Cells

**Figure 4:**
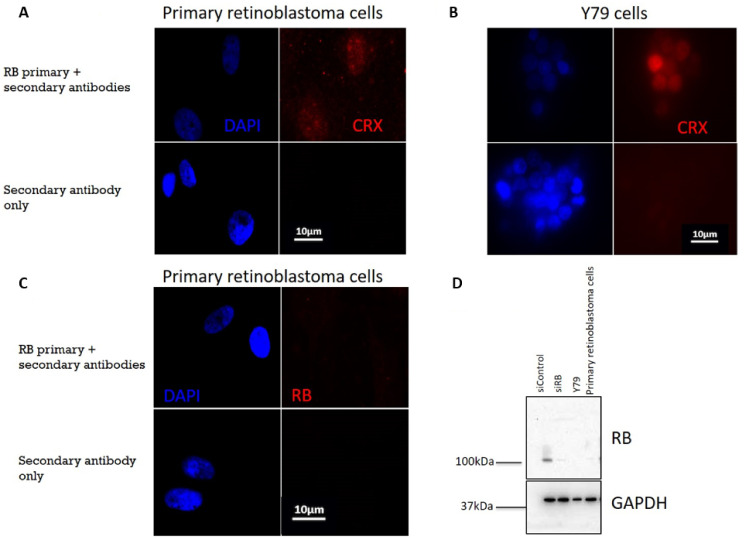
(**A**) Immunofluorescence staining detected CRX (red) in primary retinoblastoma cells. (**B**) CRX (red) was also detected in Y79 cells. (**C**) Immunofluorescence staining showed loss of RB signal (red) in primary retinoblastoma cells. Nuclei were stained with DAPI (blue). (**D**) RB was only detected in control siRNA (siControl)-treated U2OS cells. No RB could be detected in RB knocked-down (siRB) U2OS cells, Y79 cells or primary retinoblastoma cells. GAPDH was used as the loading control.

To further evaluate the treatment effects in retinoblastoma, the effects of the co-treatment of olaparib and etoposide were also studied in primary retinoblastoma cells. Primary retinoblastoma cells were isolated from an enucleated tumor from a female unilateral retinoblastoma patient diagnosed at 1 year of age. The tumor was localized in the right eye in connection with the posterior side of the lens, the ciliary body, and the iridocorneal angle. Genetic analysis by the multiplex ligation-dependent robe amplification (MLPA), DNA sequencing, and allele-specific polymerase chain reaction found that both *RB1* alleles possessed a mutation of c.470_473delins450_461 (*p*.Val157AspfsTer3). This mutation led to a deletion between position 470 and 473 and an insertion from 450 to 461 in the coding DNA sequence, resulting in an amino acid substitution valine to aspartic acid at position 157 and a stop codon termination at position 159. No *RB1* mutation was identified in the blood sample. In the primary retinoblastoma cells isolated from this patient, as well as the Y79 cells, a commercially available retinoblastoma cell line, the photoreceptor specific protein CRX expression was detectable (Figure 4A,B), confirming that these cells were developed from photoreceptors. No RB expression could be detected in the primary retinoblastoma cells and the Y79 cells (Figure 4C,D).

### 2.3. Olaparib and Etoposide Co-Treatment in Primary Retinoblastoma Cells

**Figure 5:**
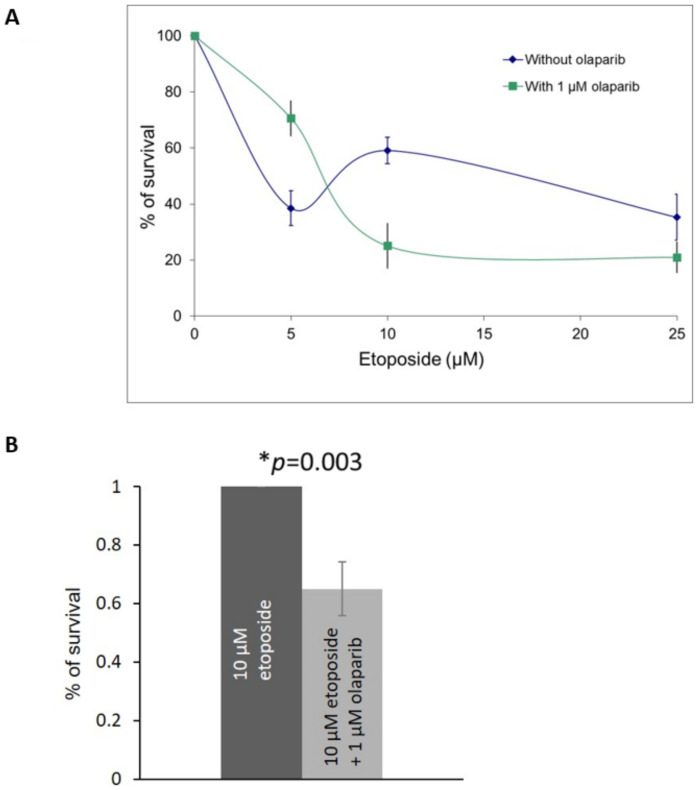
(**A**) MTT assay in primary retinoblastoma cells showed cell survival after co-treatment with 1 μM olaparib with various dosages of etoposide for three days. Experiments were repeated three times. Data are shown as mean ± standard deviation. (**B**) MTT assay in primary retinoblastoma cells showed cell survival after co-treatment with 1 μM olaparib and 10 μM etoposide for three days. Experiments were repeated three times. Data are shown as mean ± standard deviation. and analyzed using two-tailed unpaired *t*-test. * represents statistical significance *p* = 0.003.

Our results from the RB knocked-down U2OS cells demonstrated that the co-treatment of etoposide and olaparib could improve the cell killing effects. Therefore, we investigated the cell killing effects in the primary retinoblastoma cells co-treated with different dosages of etoposide in the presence of 1 µM olaparib. Enhanced killing effects could be observed in cells treated with 1 μM olaparib plus 10 μM or 25 μM etoposide (Figure 5A,B).

**Figure 6:**
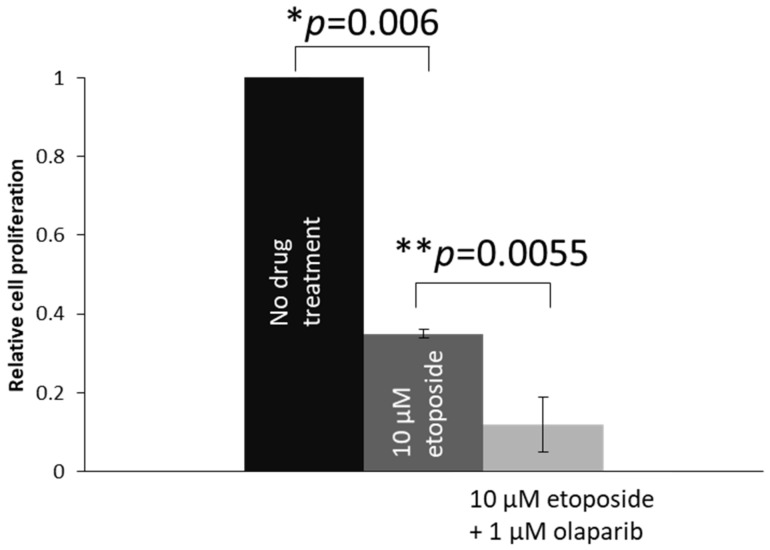
Cell proliferation analysis by using the EdU synthesis assay. Proliferation of the 1 μM Olaparib- and 10 μM etoposide-co-treated cells was significantly lower than the 10 μM etoposide-treated cells. Experiments were repeated three times. Data are shown as mean ± standard deviation and analyzed using two-tailed unpaired t test. * and ** represent statistical significance of *p* = 0.006 and *p* = 0.0055, respectively.

We also studied the effects on cell proliferation in another retinoblastoma cell line, Y79. As Y79 cells are in suspension in cell culture, EdU DNA synthesis assay was used to studied cell proliferation. Comparing Y79 cells treated with 10 µM etoposide, Y79 cells co-treated with 1 μM olaparib plus 10 μM etoposide showed significantly less proliferation (Figure 6).

## 3. Discussion

We have previously reported that RB knocked-down cells are highly dependent on MMEJ to repair DSBs. In this study, we showed that the PARP inhibitor olaparib significantly inhibited MMEJ in RB knocked-down cells by quantifying MMEJ efficiency after PARP inhibitor treatment. Importantly, in these RB knocked-down cells, we showed that the co-treatment of etoposide and olaparib could improve the cell killing effects of single-drug treatments at dosages that had no significant effects on cell survival. We further showed in the primary retinoblastoma cells that the co-treatment of etoposide with higher dosages (>10 µM) of etoposide was able to enhance cell killing, compared to the etoposide single treatment. However, a lower dose of etoposide (5 µM) did not show enhanced cell killing effects in combination with 1 µM olaparib in the primary retinoblastoma cells, while the enhanced killing effects were still observed in RB knocked-down U2OS cells. Currently, it is unclear why there were different killing effects between these two cellular systems. Possible explanations may be related to the slower cell proliferation of the primary retinoblastoma cells than U2OS cells, which doubled around 72 h and 24 h, respectively. A slower cell proliferation may contribute to less sensitivity to the co-treatment of olaparib with a low dose of etoposide. Future studies are needed to study the molecular mechanism under the co-treatment of olaparib with etoposide.

Etoposide is type II topoisomerase poison that has been widely used to treat cancer patients [19]. A PARP inhibitor is currently used to induce synthetic lethality in BRCA-deficient cancer [20]. BRCA is an important protein to promote HR. BRCA1 and 2-deficient cells also showed hypersensitivities to DSBs induced by etoposide [21]. Previous studies also showed RB functions to promote HR and C-NHEJ [15,16]. In addition, we demonstrated that RB-deficient cells are defective in HR and C-NHEJ and highly dependent on MMEJ to repair DSBs [14]. Specifically blocking MMEJ would be effective in killing retinoblastoma cells without damaging non-cancerous cells. On the other hand, PARP1 also functions to promote base excision repair (BER), which mainly repairs single strand breaks (SSBs) and protects cells against the generation of excessive SSBs during BER [22]. Besides *RB1*, there is another gene, *MYCN*, which is the most commonly amplified gene found in retinoblastoma [23]. It has been reported that a PARP inhibitor could enhance DNA replication stress and cause mitotic catastrophe in *MYCN* dependent neuroblastoma [24]. Whether the use of a PARP inhibitor is effective in treating *MYCN* mutated retinoblastoma will require further investigation. Genetically, retinoblastoma can occur in two forms: the heritable form more often results in bilateral retinoblastoma, while the non-heritable form is usually unilateral retinoblastoma. Approximately 45% of children with retinoblastoma are in the heritable form [25]. For patients possessing heritable retinoblastoma, at least one copy of the tumor initiating *RB1* mutations could also be found in other non-cancerous cells, which could lead to lower RB protein levels and higher MMEJ efficiency in these cells. If heritable retinoblastoma patients are treated systematically with a PARP inhibitor, the MMEJ pathway might also be inhibited in the non-cancerous cells, which would lead to potentially serious side effects, including synthetic lethality in these non-cancerous cells. Local PARP inhibitor treatment could be more suitable for the heritable retinoblastoma patients.

The high prevalence of secondary cancer is another serious problem for retinoblastoma survivors following recovery [26]. Whether this high secondary cancer prevalence is related to chemotherapy is not very well understood. MMEJ is a highly mutagenic pathway to repair DSBs induced by DNA-damaging drugs, such as etoposide, which can also lead to genome instability. An unstable genome would have a higher chance of accumulating further mutations that would lead to secondary cancers in retinoblastoma patients. The use of olaparib in combination with etoposide could improve the cell killing effects. Thus, lower dosages of etoposide can be used to treat retinoblastoma, which would potentially lead to lower level of DSBs and a relatively more stable genome. Further long-term animal studies are needed to evaluate the prevalence of secondary cancer after receiving the combination low-dose treatment of olaparib and etoposide.

## 4. Methods

### 4.1. Cell Culture and siRNA Transfection

Human U2OS cells were obtained from ATCC and maintained in low-glucose DMEM medium (Gibco, Amarillo, TX, USA) supplemented with 10% fetal bovine serum and 1% antibiotics (100 U/mL penicillin and 100 μg/mL streptomycin, Gibco, Amarillo, TX, USA). Cells were transfected with 100 nM control siRNA (siControl): 5′-UGGUUUACAUGUCGACUAA-3′ (Dharmacon, Lafayette, CO, USA) and *RB1* siRNA (siRB): 5′-GAAAUGACUUCUACUCGAA-3′ (Sigma-Aldrich, Burlington, MA, USA). siRNA was transfected to cells with DharmaFECT1 (Dharmacon, Lafayette, CO, USA) for 72 h. Human Y79 cells were obtained from ATCC, and primary retinoblastoma cells were isolated from the retinoblastoma tumors. The retinoblastoma tumor was cut out from the eyeball and dissociated with gentle pipetting. Primary retinoblastoma cells within the first five passages were used in this study. Both retinoblastoma cells were cultured in RPMI 1640 medium (Gibco, Amarillo, TX, USA) supplemented with 20% fetal bovine serum and 1% antibiotics (100 U/mL penicillin and 100 μg/mL streptomycin, Gibco, Amarillo, TX, USA). Both U2OS and retinoblastoma cells were maintained at 37 °C in an incubator with 5% CO_2_ and 95% air.

### 4.2. MMEJ Reporter Assay

In the reporter assay, 2.5 × 10^5^ cells were transfected with siControl and siRB for 3 days. Cells were then treated with DMSO, 1 μM or 10 μM olaparib for 1 h in both siControl- and siRB-treated cells. Cells were then transfected with the EJ2GFP (Addgene, Watertown, MA, USA) to quantify MMEJ efficiency. Furthermore, 1 μg EJ2GFP plasmids was transfected along with 1 μg pCBASceI (Addgene, Watertown, MA, USA) to cells with Fugene6 (Promega, Madison, WI, USA). The ratio of Fugene6 to the transfected plasmid was 3:1. In another round of transfection, the same numbers of cells transfected with 2 μg red fluorescent protein (RFP) expression plasmid pCAG-DsRed (Addgene, Watertown, MA, USA) were used to normalize the transfection efficiency [27]. Cells were then incubated for 72 h after transfection and collected for flow cytometry analysis.

### 4.3. MTT Cell Proliferation Assay

In the cell proliferation assay, 1.5 × 10^6^ U2OS cells were seeded in 10 cm dishes and treated with siControl or siRB for 48 h. Cells were then collected. Furthermore, 1 × 10^5^ cells were seeded into a 24-well plate. After 24 h, cells were treated with different dosages of etoposide and olaparib for three days. Cells were then incubated with 5 mg/mL MTT solution (Life technologies, Waltham, MA, USA) in 500 μL DMEM medium at room temperature for 3 h. MTT solution was then removed, and 300 μL isopropanol was added into each well. An amount of 200 μL of the mixture was transferred to a 96-well plate. Fluorescence signals were measured by a plate reader at 570 nm wavelength. To study the cell proliferation of primary retinoblastoma cells, cells were treated with etoposide and olaparib for three days, and cell proliferation was measured by MTT following the same protocol.

### 4.4. Immunofluorescence

Primary retinoblastoma cells and U2OS cells were seeded on glass coverslips. After 24 h, cells were left untreated, or treated with olaparib and etoposide for one hour. Cells were then fixed in 3.7% para-formaldehyde in phosphate buffered saline (PBS) for 10 min, washed in PBS and stored at 4 °C. After permeabilization with 0.1% TritonX-100 in PBS, cells were washed in freshly made blocking buffer (0.5 g BSA and 0.15 g glycine in 50 mL PBS). Cells were then incubated with primary antibodies against γH2AX (1:200, ab26350, Abcam, Cambridge, UK), CRX (1:200, sc-377138, Santa Cruz Biotechnology, Dallas, TX, USA) or RB antibody (1:200, ab181616, Abcam, Cambridge, UK). Cells were then incubated with donkey anti-mouse IgG AF488 (1:800, A21202, Invitrogen, Waltham, MA, USA) or donkey anti-rabbit IgG AF488 (1:800, A21206, Invitrogen, Waltham, MA, USA). The nuclei were mounted with DAPI (Vectorlabs, Burlingame, CA, USA) before coverslips were sealed to glass slides. Y79 cells were stained with the same procedures. γH2AX, CRX and RB signals were visualized with a fluorescence microscope (Nikon, Tokyo, Japan), and images were analyzed by an imaging software (SPOT Imaging Solutions, Sterling Heights, MI, USA, version 5.2).

### 4.5. Western Blotting

Cells were lysed in Laemmli sample buffer. After protein quantification by the BCA protein assay, protein samples were boiled with BB buffer (0.5M DTT, 0.25% bromophenol blue (*w*/*v*)) at 95 °C for 5 min. The denatured proteins were separated in sodium dodecyl sulfate–polyacrylamide gel and then transferred to nitrocellular membranes. Membranes were probed with primary antibodies against RB (1:500, ab181616, Abcam, Cambridge, UK) and PARP1 (1:500, MA5-15031, ThermoFisher, Waltham, MA, USA), followed by incubation with appropriate secondary antibodies coupled with horseradish peroxidase. Membranes were then incubated with the enhanced chemiluminescence substrate, and images were captured. Band intensities were quantified by using ImageJ (National Institutes of Health, Bethesda, MD, USA, version 1.52a).

### 4.6. Click-It Plus Edu Flow Cytometry Assay to Detect Cell Proliferation

In the cell proliferation assay, 4 × 10^5^ Y79 cells were seeded in a 6-well plate. After treatment with 10 μM etoposide or co-treatment with 10 μM etoposide and 1 μM olaparib for 3 days, cells were treated with 10 μM EdU for 2 h. Cells were then harvested and fixed by 100% ethanol. Cells were stained with the Click-iT Plus reaction cocktail (Life technologies, Carlsbad, CA, USA) following the manufacturer’s protocol and analyzed with flow cytometry. Relative cell proliferation was calculated by dividing the percentage of EdU positive cells in the drug-treated sample by the percentage of EdU-positive cells in the untreated sample.

## Supplementary Materials

The following are available online at https://www.mdpi.com/article/10.3390/ijms221910687/s1, Figure S1: Quantification of cells showing more than five γH2AX per cell.
