# Supplementary material for: Poly ADP Ribose Polymerase Inhibitor Olaparib Targeting Microhomology End Joining in Retinoblastoma Protein Defective Cancer: Analysis of the Retinoblastoma Cell-Killing Effects by Olaparib after Inducing Double-Strand Breaks
Source: Int J Mol Sci. 2021 Oct 1;22(19):10687. doi: 10.3390/ijms221910687 (PMC8508856; doi:10.3390/ijms221910687)
Supplement: Supplementary file 1 [file ijms-22-10687-s001.zip › ijms-1393851-supplementary.pdf]

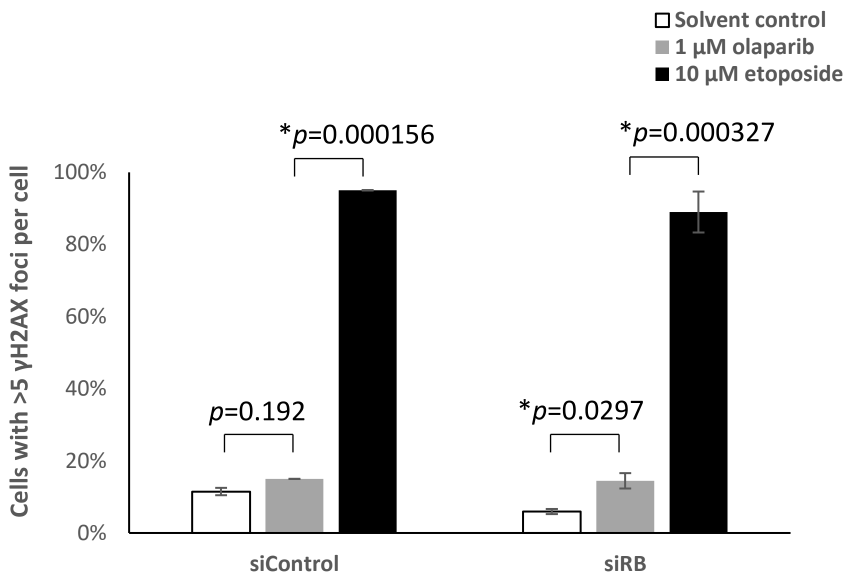

Cells showing more than five γH2AX foci per cell are quantified. Immunofluorescence staining has been repeated two times independently. Data are shown as mean ± standard deviation and analyzed using two-tailed unpaired t test. * represents statistical significance (p<0.05).

Figure S1: Quantification of cells showing more than five γH2AX per cell.
